# Characterization of septic cardiomyopathy: assessment of left ventricular diastolic function is paramount!

**DOI:** 10.1002/ehf2.14181

**Published:** 2022-09-29

**Authors:** Luigi La Via, Cristina Santonocito, Antonio Messina, Chiara Robba, Filippo Sanfilippo

**Affiliations:** ^1^ Azienda Ospedaliero Universitaria “Policlinico‐San Marco” Via Santa Sofia, 78 95123 Catania Italy; ^2^ Department of Anesthesia and Intensive Care Medicine Humanitas Clinical and Research Center‐IRCCS Via Alessandro Manzoni, 56‐20089, Rozzano Milan Italy; ^3^ Policlinico San Martino, IRCCS for Oncology and Neuroscience Genoa Italy; ^4^ Dipartimento di Scienze Chirurgiche e Diagnostiche Integrate Università di Genova Genoa Italy

*Letter on the article: Characterization of critically ill patients with septic shock and sepsis‐associated cardiomyopathy using cardiovascular MRI. Muehlberg F, Blaszczyk E, Will K, Wilczek S, Brederlau J, Schulz‐Menger J. ESC Heart Fail. 2022 May 19.*

We read with great interest the study by Muehlberg *et al*.^1^ where the authors attempted to characterize sepsis‐associated cardiomyopathy using cardiovascular magnetic resonance imaging (cMRI). In this study, 12 patients with septic shock received a cMRI study and serial transthoracic echocardiography (TTE) evaluations at 48 and 96 h. The study primarily focused on assessing LV systolic dysfunction (LVSD) by means of left ventricular ejection fraction (LVEF) and overall myocardial performance with global longitudinal strain (GLS). The authors concluded that patients with impaired LVEF have decreased GLS and impaired right ventricular systolic function; moreover, the myocardium of those with impaired LVEF function was characterized by inflammatory features (i.e. greater extracellular water volumes).^1^

We applaud the authors for their efforts in conducting such a demanding study that provides some insights in the challenge of assessing septic cardiomyopathy.^2^ However, a crucial point—the assessment of left ventricular diastolic dysfunction (LVDD)—remains not addressed at all. Of note, LVDD has been strongly associated with mortality of septic patients^3^ and with weaning failure in critically ill patients.^4^ The same association has been detected for GLS in sepsis,^5^ but not for the LVSD as evaluated by LVEF^3^ or by tissue Doppler's wave,^6^ highlighting the greater importance of LVDD over LVSD.

Considering that assessment of LVDD with cMRI has been reported in several categories of patients^7^, ^8^ and that quantification of left ventricular volumetric filling patterns may provide valuable insight into LVDD, we wonder if the authors could have information in this regard and potentially provide these analyses. Such efforts would be of significant importance as the full characterization of septic cardiomyopathy is far from being understood and addressed.

Finally, as this prospective study involves the use of TTE in critically ill patients, it may benefit from adherence to the recommendations for reporting critical care echocardiography research studies (‘Preferred Reporting Items for Critical‐care Echocardiography Studies—PRICES’),^9^, ^10^ as this may help future external validation of the study with easier between‐study comparison with similar research studies.

## Funding

None.
